# C/EBPβ Promotes Immunity to Oral Candidiasis through Regulation of β-Defensins

**DOI:** 10.1371/journal.pone.0136538

**Published:** 2015-08-28

**Authors:** Michelle R. Simpson-Abelson, Erin E. Childs, M. Carolina Ferreira, Shrinivas Bishu, Heather R. Conti, Sarah L. Gaffen

**Affiliations:** Division of Rheumatology & Clinical Immunology, University of Pittsburgh, Pittsburgh, PA, United States of America; King's College London Dental Institute, UNITED KINGDOM

## Abstract

Humans or mice subjected to immunosuppression, such as corticosteroids or anti-cytokine biologic therapies, are susceptible to mucosal infections by the commensal fungus *Candida albicans*. Recently it has become evident that the Th17/IL-17 axis is essential for immunity to candidiasis, but the downstream events that control immunity to this fungus are poorly understood. The CCAAT/Enhancer Binding Protein-β (C/EBPβ) transcription factor is important for signaling by multiple inflammatory stimuli, including IL-17. C/EBPβ is regulated in a variety of ways by IL-17, and controls several downstream IL-17 target genes. However, the role of C/EBPβ *in vivo* is poorly understood, in part because C/EBPβ-deficient mice are challenging to breed and work with. In this study, we sought to understand the role of C/EBPβ in the context of an IL-17-dependent immune response, using *C*. *albicans* infection as a model system. Confirming prior findings, we found that C/EBPβ is required for immunity to systemic candidiasis. In contrast, C/EBPβ^-/-^ mice were resistant to oropharyngeal candidiasis (OPC), in a manner indistinguishable from immunocompetent WT mice. However, C/EBPβ^-/-^ mice experienced more severe OPC than WT mice in the context of cortisone-induced immunosuppression. Expression of the antimicrobial peptide β-defensin (BD)-3 correlated strongly with susceptibility in C/EBPβ^-/-^ mice, but no other IL-17-dependent genes were associated with susceptibility. Therefore, C/EBPβ contributes to immunity to mucosal candidiasis during cortisone immunosuppression in a manner linked to β-defensin 3 expression, but is apparently dispensable for the IL-17-dependent response.

## Introduction

Oropharyngeal candidiasis (OPC, thrush) is an opportunistic infection caused by *Candida albicans*, a commensal fungus found in the skin and mucosal surfaces of most healthy individuals. Host immune responses, including locally produced cytokines and antimicrobial peptides (AMPs), prevent invasive infections by *C*. *albicans*. This microbe can also cause systemic candidiasis, a devastating nosocomial infection with a mortality rate of 40–80%. Most immunocompetent individuals maintain effective *Candida*-specific immunity, thereby keeping this fungus as a commensal microbe.

Immunosuppression, particularly that affecting the T cell compartment, triggers susceptibility to pathogenic *Candida* infections [1, 2]. In particular, OPC is highly prevalent in HIV/AIDS, with over 95% of HIV+ individuals experiencing at least one episode of oral thrush [3]. Immunosuppression with corticosteroids or broad spectrum antibiotics also enhances susceptibility of otherwise healthy individuals to *C*. *albicans* infection. Systemic candidiasis is the result of immunosuppression in combination with opportunistic exposure of *Candida* to the bloodstream, typically through medical interventions such as catheters or abdominal surgery. Fungal species are estimated to cause over 2 million infections per year worldwide with mortality rates in the range of 50%, yet there are no vaccines for *C*. *albicans*, or indeed any other fungi [4]. Therefore, it is imperative to gain a better understanding the immune response to these pathogens in order to develop more effective interventions or preventive strategies.

Recently, it has become clear that the Th17/IL-17 pathway is essential for preventing candidiasis [1]. In humans, nearly all *C*. *albicans*-reactive T helper cells are of the Th17 lineage [5]. Moreover, various syndromes that predispose humans to candidiasis converge on the Th17 or IL-17 signaling pathway. For example, neutralizing autoantibodies against Th17 cytokines are found in autoimmune polyendocrinopathy syndrome (APS-1), which is associated with chronic mucosal candidiasis (CMC) [6]. Humans with mutations in the *IL17RA* gene have been identified with CMC [7]. Similarly, mutations in *ACT1*, a key signaling intermediate in the IL-17 signaling pathway, are associated with CMC [8]. Parallel findings have been made in mice, confirming the utility of mouse models in probing underlying mechanisms of immunity to *C*. *albicans* [9].

Despite a clear role for IL-17 in mediating immunity to candidiasis, the signaling pathways used by IL-17 and its receptor in antifungal immune responses are poorly understood. Although best known for its activation of pro-inflammatory signaling pathways such as NF-κB and MAPK, IL-17 also activates transcription factors (TFs) belonging to the CCAAT/Enhancer Binding Protein (C/EBP) family [10, 11]. Many characteristic IL-17 signature genes contain C/EBP binding sites in their proximal promoters [12]. Moreover, C/EBPβ and C/EBPδ have been demonstrated to be essential for regulation of specific IL-17-induced genes, such as IL-6 and lipocalin-2 [13–15]. Regulation of C/EBPβ by IL-17 is particularly intriguing. IL-17 triggers inducible phosphorylation of C/EBPβ, which is associated with inhibition of downstream gene expression [13]. IL-17 also induces the alternative translation of C/EBPβ into multiple isoforms [16]. Both of these regulatory events are mediated through a specific C-terminal subdomain of the IL-17RA subunit [16]. To date, however, the significance of C/EBPβ in the context of IL-17 function *in vivo* is unclear.

C/EBPβ is expressed ubiquitously, and target gene activation by this TF varies among cell and tissue types. Upon activation, C/EBPβ induces a variety of genes that orchestrate immune responses, including cytokines, chemokines and their receptors. Not surprisingly, C/EBPβ^-/-^ mice are susceptible to numerous infections, including *Listeria monocytogenes* and *Salmonella typhimurium* [17, 18]. Historically, C/EBPβ has been most extensively studied in the setting of IL-1 and LPS signaling [19, 20]. In this study, we sought to understand the role of C/EBPβ in IL-17-driven immunity, using *C*. *albicans* infection as a model system. We confirmed a previous report that C/EBPβ is essential for immunity to systemic candidiasis [21]. Surprisingly and in contrast to IL-17R-deficient mice, C/EBPβ^-/-^ mice were resistant to OPC. However, under conditions of low dose cortisone-induced immunosuppression, C/EBPβ^-/-^ mice were more susceptible to OPC. This susceptibility correlated with expression of β-defensin 3, but not with other IL-17-dependent genes.

## Materials and Methods

### Mice

*Cebpb* ^*tm1Vpo*^/J^+/+^ mice (The Jackson Laboratory, Bar Harbor ME) were bred to generate experimental animals, with sample sizes based on power analyses calculated from previously published data [9]. Genotypes were verified for all mice by PCR of ear biopsies. C57BL/6J mice (The Jackson Laboratory) were used for cortisone titrations. All mice weighed approximately 20g and were housed under a 12 hour light/dark cycle in SPF conditions with autoclaved food and water *ad libitum*. Cohorts were selected randomly and were age- and sex-matched using both males and females at an age range of 6–10 weeks. Mice were monitored visually and weighed at least once daily after infection. The University of Pittsburgh Institutional Animal Care and Use Committee (IACUC) approved all animal protocols used in this study (Animal welfare assurance number: A3187-01). All efforts were made to minimize suffering, in accordance with recommendations in the Guide for the Care and Use of Laboratory Animals of the National Institutes of Health.

### Oral candidiasis

Mice were pre-swabbed orally prior to each experiment to verify the absence of pre-existing *Candida* colonization. Mice were inoculated sublingually for 75 mins under anesthesia (ketamine 100 mg/kg and xylazine 10 mg/kg injected i.p.) with *C*. *albicans* (strain CAF2-1) placed in an sterile saturated 0.0025 mg cotton ball, as described [22, 23]. At the end of the time course (5 d), tongue tissue was homogenized using a Miltenyi GentleMacs Dissociator (Miltenyi Biotec). Half the tissue was prepared for mRNA analysis or histology. The other half was serially diluted and plated in triplicate on YPD agar and colony-forming units (CFU) enumerated for tissue fungal burden determination. Mice were weighed daily. Mice were sacrificed for humane reasons by CO_2_ inhalation if they lost more than 25% weight loss or exhibited signs of pain or distress as delineated by the approved animal protocol. There were no severe adverse events in any group.

### Disseminated Candidiasis

*C*. *albicans* (strain CAF2-1) was grown overnight in YPD at 30°C. Age- and sex-matched mice were injected in the tail vein with 1-2x10^5^ *C*. *albicans* cells in 100ul PBS, as described [9, 24]. For injections, mice were briefly held in a commercial restraining apparatus (Braintree Scientific, Braintree MA). Mice were humanely sacrificed by CO_2_ inhalation followed by cervical dislocation after 10 d, weight loss exceeded 20% or when animals exhibited signs of distress such as severe hunching, shivering or loss of righting. There were no unexpected adverse events in any group.

### Cell Culture

OKF6/TERT2 cells [25] were provided by J. Rheinwald (Brigham & Women’s Hospital, Boston MA) cultured in Serum-Free Fibroblast media, 25 ug/ml Bovine Pituitary Extract and 2 ug/ml EGF (Life Technologies, Grand Island NY). A heat killed (HK) extract of *C*. *albicans* (strain CAF2-1) was prepared with ~ 4×10^8^ yeast cells boiled for 45 mins. OKF6/TERT2 cells were treated with 200 ng/ml IL-17 and 2ng/ml TNFα or 2×10^6^ HK *C*. *albicans* for 24 hours.

### Real-time RT-PCR

RNA from tongue was extracted with RNAeasy Kits and cDNA synthesized with a SuperScript III First-Strand Synthesis System (Invitrogen). Gene expression was determined by qPCR with PerfeCTa SYBR Green FastMix ROX (Quanta BioSciences) on a 7300 Real-Time PCR System (Applied Biosystems), normalized to *Gapdh*. Primers were from Quantitect (Qiagen).

### Histology

Tongue tissue was prepared for histology by the Research Histology Services core of the University of Pittsburgh. Samples were stained with periodic acid Schiff (PAS) or H&E and imaged at 10–40X magnification.

### Statistics

A minimum of two replicates were performed for all experiments unless noted. Data were compared by ANOVA and Mann-Whitney correction or unpaired Student’s t-test using Graphpad Prism (v. 4). *P* values <0.05 were considered significant.

## Results

### C/EBPβ-deficient mice are susceptible to disseminated candidiasis

**Fig 1:**
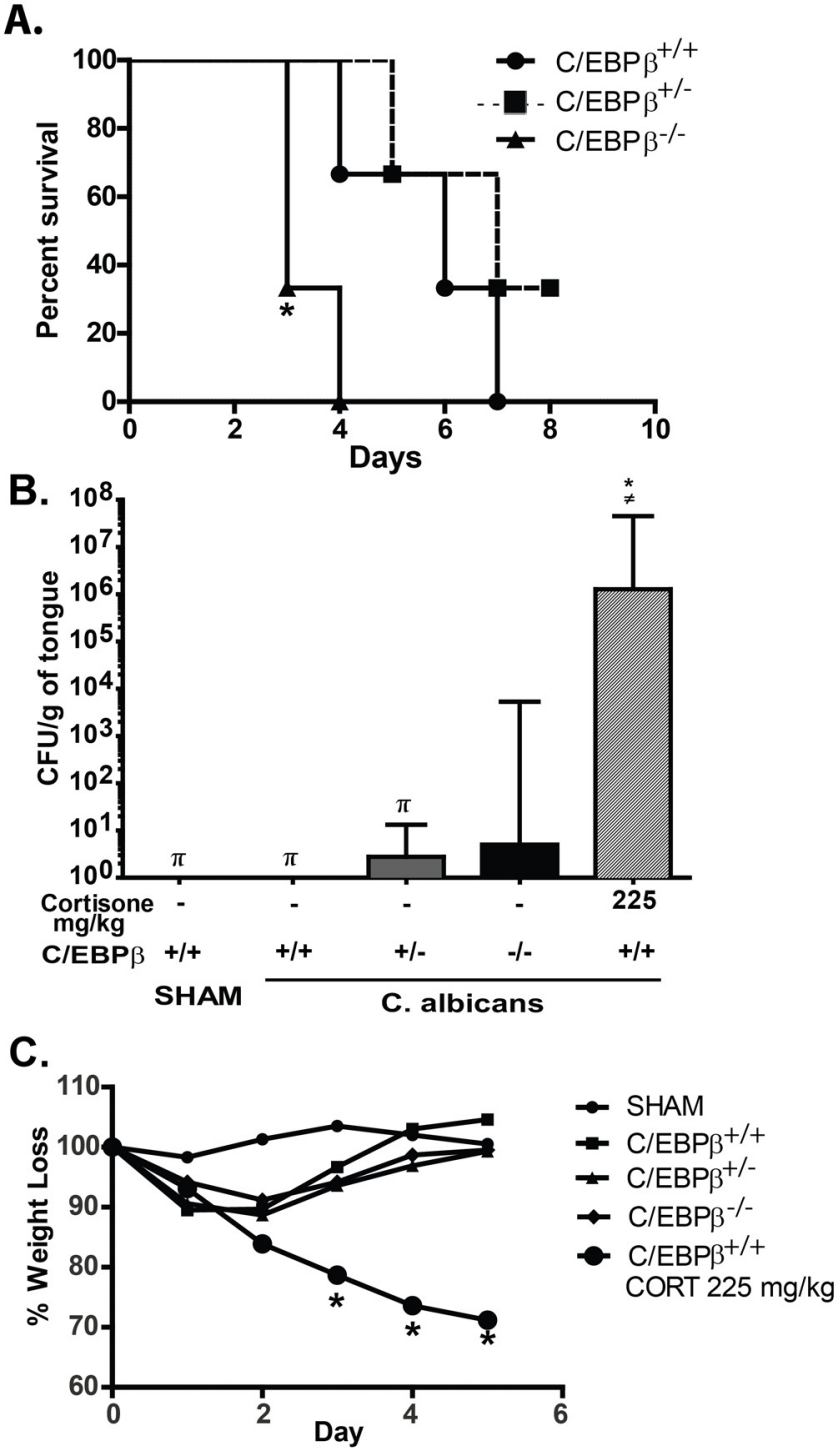
C/EBPβ^-/-^ mice exhibit increased susceptibility to systemic candidiasis but are resistant to oral candidiasis. (**A**) The indicated mice (n = 3 per group) were injected with *C*. *albicans* in the lateral tail vein. Time to sacrifice is indicated (days). *P<0.05 versus (vs) C/EBPβ^+/-^ mice using Log-rank (Mantel-Cox). (**B**) The indicated mice were infected sublingually with *C*. *albicans* for 75 mins. After 5 d, fungal loads in tongue were assessed by CFU enumeration of tongue tissue homogenates. Bars indicate geometric mean with 95% CI. C/EBPβ^+/+^ SHAM (n = 3), C/EBPβ^+/+^ (n = 5), C/EBPβ^+/-^ (n = 9), C/EBPβ^-/-^ (n = 3), C/EBPβ^+/+^ plus 225mg/kg cortisone (n = 3); cortisone acetate was administered by subcutaneous injection on days -1, +1 and +2 relative to infection. *P*<0.05 by t-test with Mann-Whitney correction: * vs C/EBPβ^+/+^ WT, ≠ vs C/EBPβ^+/-^, # vs C/EBPβ^-/-^, π vs C/EBPβ^+/+^ 225mg/kg. **C**. Weights of mice were assessed daily and graphed as percent of starting weight. * vs C/EBPβ^+/+^ WT. *P*<0.05 by t-test with Mann-Whitney correction. Experiment was performed once.

To evaluate the role of C/EBPβ in antifungal immunity, we subjected C/EBPβ^-/-^ mice to disseminated candidiasis, the most commonly-employed model of *C*. *albicans* infection [9]. Systemic candidiasis was induced with an intravenous inoculum of 2x10^5^ *C*. *albicans* yeast cells. Because C/EBPβ^-/-^ mice are on a mixed genetic background, littermates were used as controls in these and all subsequent experiments [21]. It should be noted that C/EBPβ^-/-^ mice are notoriously difficult to generate, with homozygous offspring surviving at far below expected Mendelian ratios. Nonetheless, we were able to generate animals for experimentation, although their numbers were of necessity lower than littermate controls. As expected, C/EBPβ^-/-^ mice infected intravenously with *C*. *albicans* succumbed to disease by day 4 post-infection, whereas C/EBPβ^+/+^ and C/EBPβ^+/-^ mice survived beyond day 7 (Fig 1A). These data are consistent with documented susceptibility of the C/EBPβ^-/-^ mice to other infections [18], and confirm published data in systemic candidiasis [21].

### C/EBPβ-deficient mice are susceptible to oral candidiasis under conditions of immunosuppression

The most common form of candidiasis in humans occurs at mucosal surfaces, particularly in the oral cavity. Considerable evidence supports a vital role for IL-17 in immunity to OPC [26], and IL-17 induces downstream signals in part through C/EBPβ [13, 14]. Therefore, to determine the extent to which C/EBPβ-dependent pathways drive immunity to OPC, we subjected C/EBPβ^-/-^ mice to OPC. In the standard mouse model of OPC, WT mice are resistant to infection, clearing the fungus within 4–5 days and exhibiting no overt symptoms of thrush. In contrast, mice subjected to high dose cortisone exhibit severe OPC symptoms, including weight loss, visible fungal lesions on the tongue and buccal mucosa, and a high fungal load determined by CFU assessment of homogenized tongue tissue plated on YPD agar [27]. The control mice behaved as expected, with C/EBPβ^+/+^ and C/EBPβ^+/-^ mice fully clearing *C*. *albicans* from the oral cavity by day 5 post infection. High-dose cortisone treatment (225 mg/kg) caused severe susceptibility to OPC, with fungal loads averaging 1.2x10^6^ CFU/g. Unexpectedly, C/EBPβ^-/-^ mice were fully resistant to infection, exhibiting weight loss profiles and oral CFU levels similar to C/EBPβ^+/+^ and C/EBPβ^+/-^ control mice, in the range of 1–5 CFU/g tongue tissue (Fig 1B and 1C). Therefore, OPC is to our knowledge the first infection to which C/EBPβ^-/-^ mice are resistant. In contrast, we previously showed that IL-17RA^-/-^, IL-17RC^-/-^ and Act1^-/-^ mice are susceptible to OPC [23, 28, 29]; therefore, these data further demonstrate that C/EBPβ appears to be dispensable for an effective IL-17-dependent immune response.

**Fig 2:**
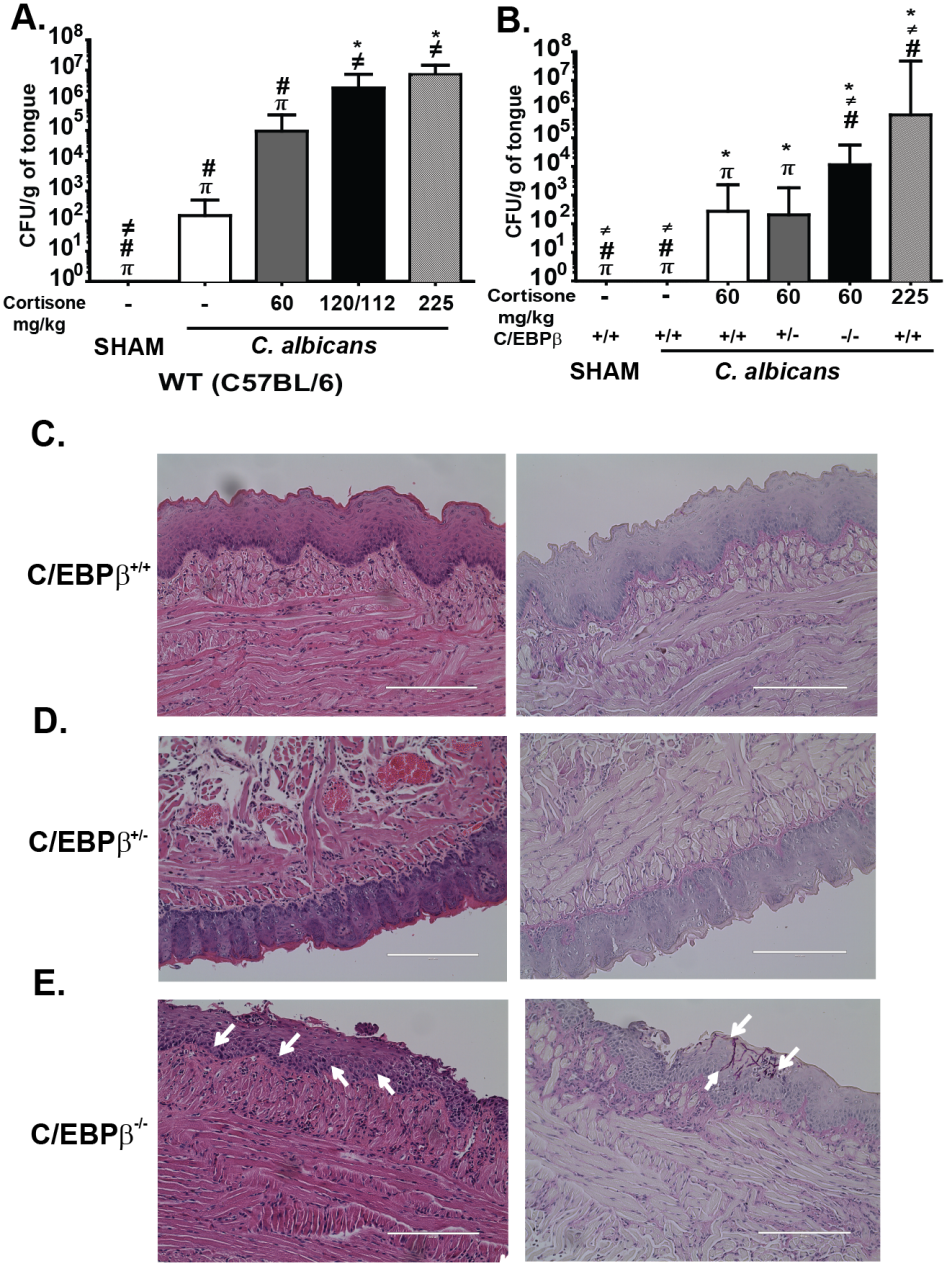
C/EBPβ^-/-^ mice exhibit increased susceptibility to oral candidiasis in the context of cortisone-induced immunosuppression. **(A)** C65BL/6 mice (“WT”) were treated with the indicated doses of cortisone acetate at days -1, +1 and +2 relative to infection. After 5 d, fungal loads in tongue were assessed by CFU enumeration of tongue tissue homogenates. SHAM (n = 3), No cortisone control (n = 5), 60mg/kg (n = 9), 120/112mg/kg (n = 8), and 225mg/kg (n = 8). Data are pooled from 2 independent experiments. Bars indicate geometric mean with 95% CI. P<0.05 by t-test with Mann-Whitney correction: * vs NO CORT, ≠ vs 60mg/kg, # vs 120/112mg/kg and π vs 225mg/kg. (**B**) The indicated mice were infected orally as described in panel A. Cortisone acetate was administered subcutaneously on days -1, +1 and +2 relative to infection. C/EBPβ^+/+^ SHAM (n = 3), C/EBPβ^+/+^ (n = 5), C/EBPβ^+/+^ 60 mg/kg (n = 16), C/EBPβ^+/-^ 60mg/kg (n = 16), C/EBPβ^-/-^ 60mg/kg (n = 10) and C/EBPβ^+/+^ 225mg/kg (n = 4). *P*<0.05 by t-test with Mann-Whitney correction: * vs C/EBPβ^+/+^ NO CORT, ≠ vs C/EBPβ^+/+^ 60mg/kg, # vs C/EBPβ^+/-^ 60mg/kg and π vs C/EBPβ^-/-^ 60mg/kg. Data are pooled from two independent experiments. **C.** Representative tongue sections from the indicated mice were stained with H&E or Periodic-acid Schiff (PAS). Scale bar indicates 200 μM. White arrows indicate hyphae.

Since the C/EBPβ^-/-^ mice did not develop OPC upon infection with *C*. *albicans*, a plausible alternative hypothesis was that they might instead exhibit enhanced resistance to infection. Indeed, C/EBPβ has been linked to inhibition of IL-17-mediated signal transduction *in vitro* in several prior studies [13, 16]. However, to address this question, we needed to establish a model system in which WT mice exhibited a mild degree of disease so that we could observe enhanced resistance to OPC. Accordingly, WT mice were treated with graded doses of cortisone acetate and infected orally with *C*. *albicans*. This regimen resulted in a dose-dependent susceptibility to OPC (Fig 2A). Based on these data, we selected 60 mg/kg as an appropriate concentration, as mice given this dose exhibited an average of 89 CFU/g tongue. Accordingly, C/EBPβ^-/-^ or control mice were subjected to OPC in combination with 60 mg/kg cortisone acetate. As shown, C/EBPβ^-/-^ mice given 60 mg/kg cortisone showed an average CFU of 11,395 CFU/g, which was significantly greater than fungal loads in the C/EBPβ^+/+^ and C/EBPβ^+/-^ mice given cortisone (average loads = 271 and 203 CFU/g, respectively) (Fig 2B). As expected, high dose cortisone (225 mg/kg) caused even higher fungal burdens (6x10^5^ CFU/g). Invasive hyphae were observed in PAS-stained tongue sections from C/EBPβ^-/-^ mice but not in controls (Fig 2C). These data indicate that C/EBPβ contributes to immunity to OPC, but only under conditions of cortisone-mediated immunosuppression.

### Mechanisms of C/EBPβ-mediated immunity to OPC

**Fig 3:**
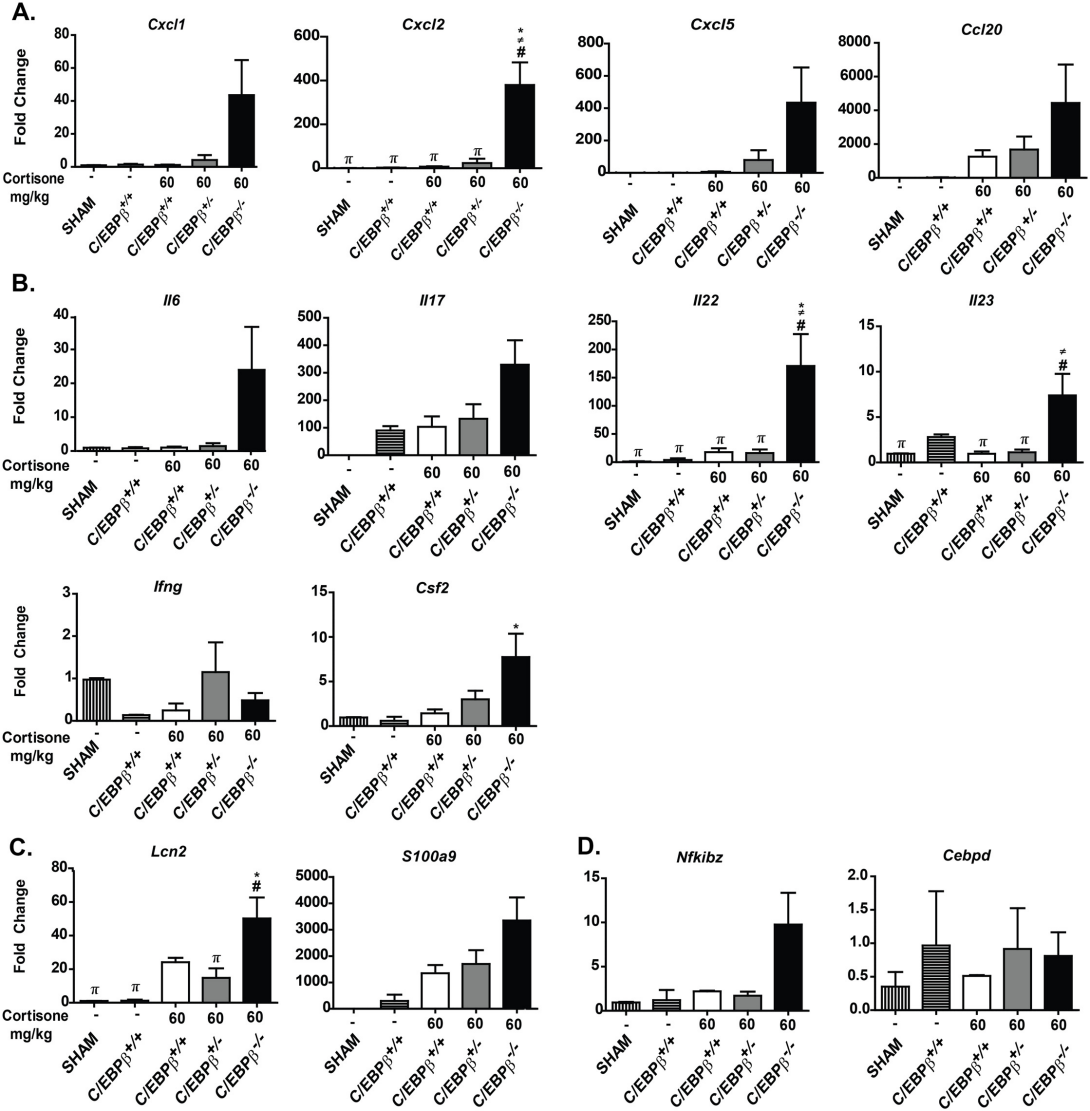
The susceptibility of C/EBPβ^-/-^ mice to OPC does not correlate with expression of prototypical IL-17-regulated genes. mRNA from tongue was isolated from the indicated mice 5 days after oral *C*. *albicans* infection [C/EBPβ^+/+^ SHAM (n = 3), C/EBPβ^+/+^ NO CORT (n = 3), C/EBPβ^+/+^ 60mg/kg (n = 5), C/EBPβ^+/-^ 60mg/kg (n = 6), and C/EBPβ^-/-^ 60mg/kg (n = 5)]. Complementary DNA was prepared and subjected to qPCR analysis to detect the indicated genes. Results are presented as fold induction over SHAM treated mice and normalized to expression of *Gapdh*. Data are pooled from 2 independent experiments. *P*<0.05 by student unpaired t-test: * vs C/EBPβ^+/+^ NO CORT, ≠ vs C/EBPβ^+/+^ 60mg/kg, # vs C/EBPβ^+/-^ 60mg/kg and π vs C/EBPβ^-/-^ 60mg/kg.

In order to understand the mechanisms by which C/EBPβ contributes to immunity to OPC, we evaluated expression of genes previously shown to be induced in immunocompetent mice during oral infection with *C*. *albicans* [23, 30]. To that end, we measured mRNA levels of chemokines, cytokines anti-microbial peptides and TFs in the oral mucosa (tongues) of mice subjected to OPC, measured at day 5. We were specifically seeking genes that showed an impairment in C/EBPβ^-/-^ mice compared to controls. Somewhat surprisingly, expression of most genes was not impaired in infected C/EBPβ^-/-^ mice; in fact, in many instances, transcripts were expressed at higher levels compared to controls (Fig 3, Table 1). These genes included chemokines (*Cxcl1*, *Cxcl2*, *Cxcl5*, Fig 3A), cytokines (*Il6*, *Csf3*, *Il17a*, *Il22*, Fig 3B), antimicrobial peptides (*Lcn2*, *S100a8*, Fig 3C) and immunoregulatory transcription factors (*Nfkbiz*, *Cepbd*, Fig 3D). The elevated expression of these factors in C/EBPβ^-/-^ mice was somewhat unexpected, but probably reflects the increased fungal burden in the mice at this time point with a concomitant active immune response.

**Table 1:** (Simpson-Abelson *et al*.).

*Gene*	Protein	*C/EBPβ Genotype*		
		+/+	+/-	-/-
*Il6*	IL-6	-	-	***
*Il17a*	IL-17A	****	****	****
*Il22*	IL-22	***	***	****
*Il23*	IL-23p19	-	-	*
*Csf2*	G-CSF	-	*	*
*Ifng*	IFNγ	-	-	-
*Cxcl1*	CXCL1/KC	-	*	***
*Cxcl2*	CXCL2	*	***	****
*Cxcl5*	CXCL5	*	***	****
*Ccl20*	CCL20	****	****	****
*S100a9*	S100A9	****	****	****
*Lcn2*	Lipocalin-2/24p3	*	*	***
*Defb3*	β-defensin 3	***	***	**
*Nfkbiz*	IκBζ	*	*	**
*Cebpd*	C/EBPδ	-	-	-

Transcript expression levels of the indicated genes in tongues isolated from the indicated mice 5 days after induction of oropharyngeal candidiasis. Increase over Sham-infected mice is indicated as follows:

*2-4-fold,

** 5–10 fold,

*** 10–50 fold,

**** > 100-fold

**Fig 4:**
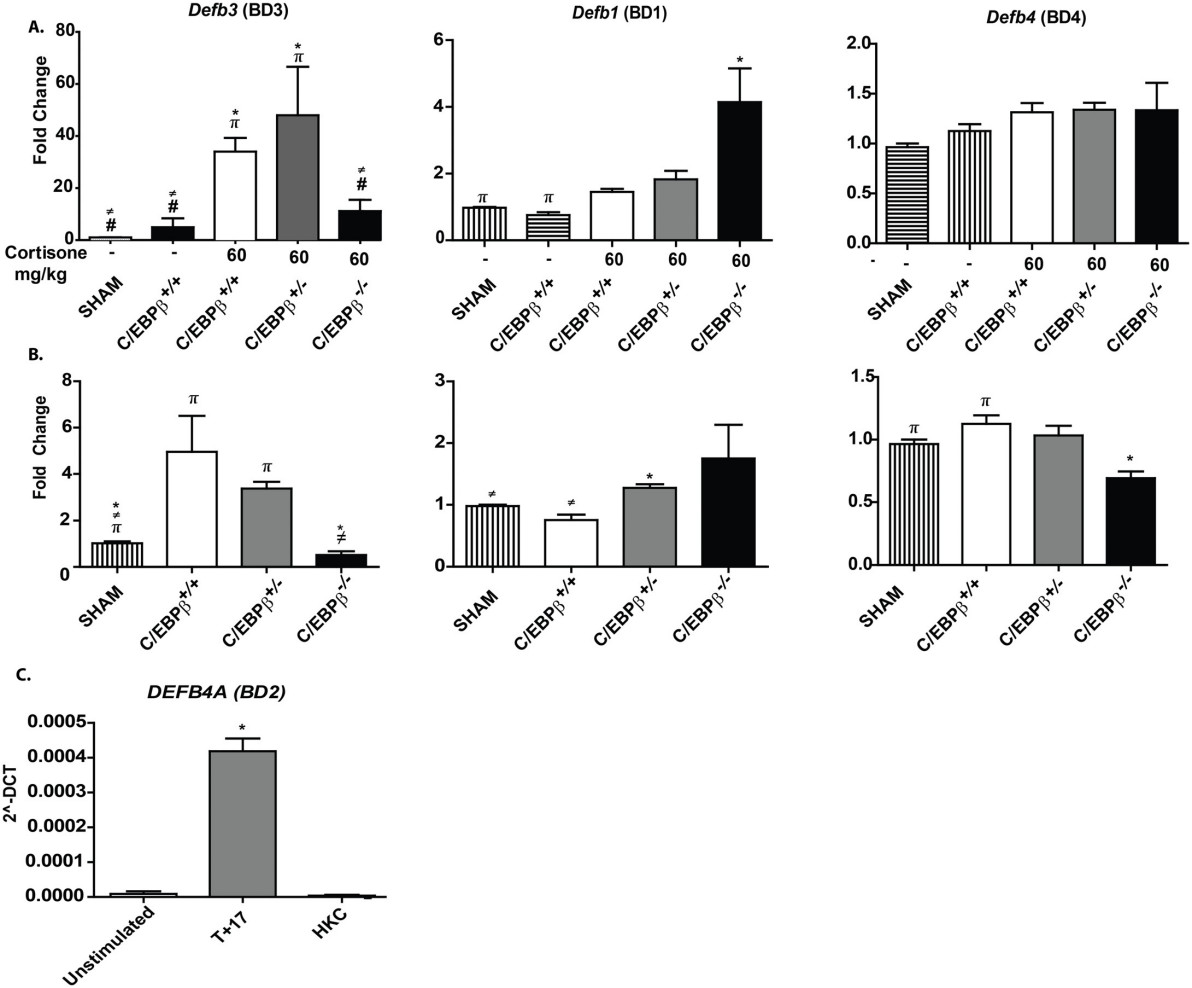
Susceptibility of C/EBPβ^-/-^ mice to OPC correlates with expression of BD3. **(A)** mRNA from tongue was isolated from the indicated mice 5 days after oral *C*. *albicans* infection [C/EBPβ^+/+^ SHAM (n = 3), C/EBPβ^+/+^ NO CORT (n = 3), C/EBPβ^+/+^ 60mg/kg (n = 5), C/EBPβ^+/-^ 60mg/kg (n = 6), and C/EBPβ^-/-^ 60mg/kg (n = 5)]. Complementary DNA was prepared and subjected to qPCR analysis to detect the indicated genes. Results are presented as fold induction over SHAM treated mice and normalized to expression of *Gapdh*. Data are pooled from two independent experiments. *P*<0.05 by student unpaired t-test. * vs C/EBPβ^+/+^ NO CORT, ≠ vs C/EBPβ^+/+^ 60mg/kg, # vs C/EBPβ^+/-^ 60mg/kg and π vs C/EBPβ^-/-^ 60mg/kg. (**B)** mRNA from tongue was isolated from the indicated mice 5 days after oral *C*. *albicans* infection and analyzed as in panel A. C/EBPβ^+/+^ SHAM (n = 3), C/EBPβ^+/+^ (n = 2), C/EBPβ^+/-^ (n = 2), and C/EBPβ^-/-^ (n = 2). Data are from one experiment. * *P*<0.05 by student unpaired t-test. vs C/EBPβ^+/+^, ≠ vs C/EBPβ^+/-^ and π vs C/EBPβ^-/-^. (**C)** OKF6/TERT2 human oral keratinocytes were treated with 200 ng/ml IL-17 plus 2ng/ml TNFα or with 2×10^6^ HK *C*. *albicans* for 24 h. Complementary DNA was prepared and subjected to qPCR analysis to detect *DEFB4A*. Data are normalized to expression of *GAPDH* and represent absolute levels. Data are representative of 2 independent experiments. *P<0.05 compared to unstimulated OKF6/TERT2 cells.

In contrast to the aforementioned genes, the expression of *Defb3* did correlate with susceptibility to OPC, as expression was much lower in C/EBPβ^-/-^ mice compared to littermate controls (Fig 4A). *Defb3* encodes the antimicrobial peptide β-defensin 3 (BD3), which is the accepted murine homologue of human BD2. BD3 expression has been previously linked to IL-17-immunity to OPC, and this AMP also has intrinsic, direct candidacidal activity [23, 30, 31]. In light of this observation, we also assessed the expression of Defb3 in non-immunosuppressed settings. Although *Defb3* was induced in C/EBPβ^+/+^ and C/EBPβ^+/-^ mice, expression was impaired in C/EBPβ^-/-^ animals (Fig 4B). However, overall expression was notably lower than in cortisone-treated mice (Fig 4A and 4B). Therefore, impaired *Defb3* expression correlates with C/EBPβ-deficiency in cortisone-induced susceptibility to OPC, and may account for the increased fungal burdens seen in these conditions.

Based on this observation, we assessed other members of the β-defensin family that have been linked to candidiasis, namely *Defb1* and *Defb4* [32–35]. However, neither showed the correlative link to C/EBPβ-mediated disease susceptibility that was seen for *Defb3* (Fig 4A). We also assessed *Defb2*, but expression was undetected in this tissue (data not shown). Modest changes were also seen for these genes in non-immunosuppressed conditions (Fig 4B), but these differences were so slight we think it unlikely that they contribute to the susceptible phenotype.

Finally, to determine whether the impact of C/EBPβ could be isolated to oral epithelial tissue, we asked whether *C*. *albicans* exposure could induce this defensin directly in oral keratinocytes. There is a paucity of tractable murine oral epithelial cell systems in which to assess this issue, so instead we used the human OKF6/TERT2 oral keratinocyte cell line [25]. Cells were cultured for 24 with a heat-killed (HK) *C*. *albicans* cell extract [30]or with inflammatory cytokines (IL-17+ TNFα) as a positive control. We assessed expression of *DEFB4A*, which encodes BD2, the human orthologue of murine BD3. Whereas cytokines induced substantial expression of DEFB4A, there was no detectable induction of this gene upon C. albicans treatment, which held true at both early and late time points (Fig 4C, data not shown).

## Discussion

Since IL-17 is a key regulator of immunity to oral candidiasis and mediates gene regulation through C/EBPβ, the impetus for this study was to understand possible connections between IL-17 signaling, C/EBPβ and antifungal immunity. The C/EBP transcription factors are central regulators of immune responses, controlling expression of a myriad of cytokines, receptors and other genes important in host defense against infection [20]. C/EBPβ [also known as liver activated protein (LAP) or nuclear factor inducing IL-6 (NF-IL6)] is an intronless gene that was among the first TFs to be characterized, yet is surprisingly poorly understood in the context of mammalian immunity. In part this deficit is due to the early lethality of C/EBPβ^-/-^ mice. C/EBPβ^-/-^ animals often die shortly after birth, an effect that is more pronounced in non-SPF conditions [21]. These mice exhibit lymphoproliferative and myeloproliferative disorders and have high circulating levels of IL-6 [18]. Not surprisingly, C/EBPβ^-/-^ mice are highly sensitive to many pathogens, including *Listeria monocytogenes*, *Salmonella typhimurium* as well as systemic *C*. *albicans* (Fig 1A) [18, 21].

C/EBPβ is subject to numerous forms of post-transcriptional and post-translational modifications, many of which are regulated by inflammatory stimuli such as LPS, IL-1 and IL-17. For example, C/EBPβ undergoes alternative translation into three functionally distinct isoforms, with IL-17 enhancing expression of the LAP* and liver inhibitory protein (LIP) isoforms relative to the normally dominant LAP isoform [16, 36]. IL-17 also induces phosphorylation of C/EBPβ on sites that are known to regulate its transcriptional capacity [13, 37]. Given these connections, we were surprised to observe that C/EBPβ-deficient mice were fully resistant to OPC, at least in the absence of underlying immunosuppressive drugs. In fact, as far as we know, OPC is the only infectious disease to which these mice have been found to be resistant (Fig 1B).

IL-17 deficiency causes mucosal candidiasis in humans with rare genetic defects in this pathway [38]. However, a much more common risk factor for OPC in the general population is the use of immunosuppressive drugs, including systemic or inhaled corticosteroids. Exactly why this is the case is poorly understood at the molecular level, but our data suggest that C/EBPβ is an important component of oral immunity in this context. The gene encoding CEBPβ is upregulated in human epithelial and endothelial cells in response to *Candida* exposure as well as in samples from women with vaginal candidiasis [39]. Moreover, our data imply that this susceptibility is not due to a failure of IL-17 signaling, since C/EBPβ^-/-^ mice are resistant to OPC, whereas mice with impaired IL-17 signaling capability (e.g., IL-17RA^-/-^, IL-17RC^-/-^, Act1^-/-^ or RORγt^-/-^ mice) are all highly susceptible [23, 28, 29, 40]. Notably, IL-17R-deficient mice have significantly lower oral fungal burdens of *C*. *albicans* than mice given high dose corticosteroids [23], indicating that C/EBPβ appears to contribute to the non-IL-17-dependent arm of antifungal immunity. One mechanism by which C/EBPβ contributes to cortisone-induced OPC may be in regulating the monocyte/macrophage lineage. C/EBPβ is potently induced by LPS and TNFα in these cells, where it regulates a myriad of genes controlling proliferation, differentiation and function of these innate cells [41]. Consistently, depletion of monocytes and neutrophils induces severe OPC in mice, with fungal loads similar to cortisone-treated animals and several logs higher than IL-17RA-deficient mice [42].

Our data suggest a link between C/EBPβ and *Defb3* gene expression in OPC, but not other classic IL-17 target genes that are regulated by C/EBPβ such as *Il6* or *Lcn2* [12] (Table 1, Fig 4). Indeed, neither IL-6^-/-^ nor Lcn2^-/-^ mice are susceptible to OPC [29, 40], which is consistent with the resistance of C/EBPβ^-/-^ mice to this disease. In this regard, β-defensins are well established as regulators of immunity to *C*. *albicans* and other mucosal pathogens [43]. BD3 and its human orthologue BD2 are expressed in oral mucosa during exposure to *C*. *albicans* [23, 30, 44–46]. Upregulation of BD2 in lung epithelial cells is regulated by IL-17 and other cytokines through the NF-κB and PI3K pathways [47–49]. It is likely that similar pathways are operative in oral epithelium as well. In addition to its activity as an antimicrobial peptide, BD3 is also a ligand for the chemokine receptor CCR6, which is expressed on various mucosal lymphocytes including IL-17-expressing cells [31, 50]. We speculate that both activities are operational in the setting of OPC, but of course further experimentation in this regard is warranted.

A complete profile of genes regulated by C/EBPβ during cortisone-induced OPC would require a global analysis such as ChIP-Seq, which would be technically challenging since it is difficult to purify enough viable oral epithelial cells from tongue to accomplish such a study [23, 51]. This is especially true following steroid use, which depletes hematopoietic cell numbers. In attempting to identify a system where a more molecular approach might be accomplished, we analyzed a human oral keratinocyte cell line, OKF6/TERT2 [25] for the ability to induce BD2 (Fig 4C). Although the gene encoding BD2 was induced in response to inflammatory cytokines (IL-17 and TNFα), treatment with a heat-killed *C*. *albicans* extract alone did not induce this defensin. Since the impact of C/EBPβ deficiency seems to be largely independent of IL-17, this system is not suitable for understanding how BD2 (and by extension, BD3) is regulated by C/EBPβ in this setting.

In summary, these data shed new light on how immunity to mucosal candidiasis is controlled, and also the contribution of C/EBPβ to steroid-induced fungal susceptiblity. Defensins may therefore be an attractive therapeutic target in treating mucosal fungal infections associated with immunosuppression.
